# Groundwater quality assessment for different uses using various water quality indices in semi-arid region of central Tunisia

**DOI:** 10.1007/s11356-020-11149-5

**Published:** 2020-10-29

**Authors:** Soumaya Aouiti, Fadoua Hamzaoui Azaza, Fetheddine El Melki, Monji Hamdi, Fulvio Celico, Mounira Zammouri

**Affiliations:** 1 Faculty of Sciences of Tunis, Sedimentary Environments, Oil Systems and Reservoir Characterization Laboratory, University of Tunis El Manar, UR11 ES15, 2092 Tunis, Tunisia; 2 Department of Chemistry, Life Sciences and Environmental Sustainability, University of Parma, Parco Area delle Scienze 157a, 43124 Parma, Italy; 3 Faculty of Sciences of Tunis, Geodynamics, Geonumerics and Geomaterials Laboratory, University of Tunis El Manar, Lab3G, 2092 Tunis, Tunisia; 4 Commissariat Régional au Développement Agricole (CRDA), Sidi Bouzid, Tunisia

**Keywords:** Drinking and irrigation suitability, Quality, WHO, Tunisian standard, Quality indices, Hajeb Layoun-Jelma basin

## Abstract

The Hajeb Layoun-Jelma basin, located in the central Tunisia, is the principal source of water supply for Sidi Bouzid and Sfax region. The over-abstraction from this groundwater, since 1970, and the intensive agriculture activities led to the degradation of the water quantity and quality. The quality evaluation for this groundwater is very important tool for sustainable development and decision for water management. A total of 28 groundwater samples, from shallow, springs, and deep aquifers, were collected, storage and analyzed to evaluate its quality suitability for domestic and agriculture purposes using geographic information system and geochemical methods. For the both aquifers, the abundance of cations: Na > Mg > Ca > K, and of anions in the order: Cl > HCO_3_ > SO_4_. The dominant hydrochemical facies, for the shallow aquifer and springs, are Na-Cl and Ca-Mg-Cl; for the deep aquifer, the geochemical facies are Na-Cl, Ca-Mg-Cl, and Ca-Cl. The comparison of the major parameters and the chemical data with the World Health Organization standards and the national standards indicate that this groundwater is suitable for drinking, except in some samples, with high salinity concentrations. The water quality was assessed, for drinking uses, using “water quality index,” “entropy,” and “improved water quality index.” The results mentioned that the improved water quality index is the best method which indicated that the poor water quality coincide with the Na-Cl water type. The entropy method and the water quality index present the optimistic methods. The irrigation suitability assessment was made using various parameters (SAR, TH, % Na, PI, MH, KR, EC). The results revealed that the majority of samples in Hajeb Layoun-Jelma basin are not appropriate for irrigation uses.

## Introduction

Water is the principal component in the earth which supports the life of all living. Groundwater is a very important source of water, specifically in the semi-arid and arid region. It supports all types of uses (drinking, irrigation, and industrial) (Hamzaoui-Azaza et al. 2020). However, groundwater is threatened by severe problems caused by natural/anthropogenic factors, such as the extensive agricultural activities, the marine intrusion, the population growth, and the industrial development (Zammouri et al. 2013). This factor engendered a degradation in the quality and the quantity of groundwater in many countries: for example, Ameur et al. (2016) found that the water quality, in the northeast Tunisia, is at poor level due to the nitrate pollution that originate from the excessive use of nitrate-rich fertilizers. Adimalla (2019) conducted a study on the effect of the rapidly urban activities (South India) on water quality and the human health risk related to the nitrate and the fluoride pollution. Mnassri et al. (2018) demonstrate that the sources of the groundwater salinization (central-eastern Tunisia), which the salinity exceeding 6 g L^−1^, originate from an anthropogenic/natural factors (dissolution of halite, precipitation of carbonate coupled with the dissolution of gypsum, evaporation, and intensive irrigation practices), and Ligavha-Mbelengwa and Gomo (2020) conducted a work investigated of factors influencing the water quality (South Africa), and it indicated that both anthropogenic and natural factors are controlling the groundwater quality of this site.

The water quality has a strong relation with the health risk (Ricolfi et al. 2020); for this, the water quality evaluation is very important and widely studied in many regions around the world (Barbieri et al. 2019; Su et al. 2019; Asadi et al. 2020).

Various methods are used for the water quality evaluation; for drinking uses, we cited the following: the “Water quality index” (WQI) (Ghouili et al. 2018), “the Entropy water quality index” (Islam et al. 2017), “the improved water quality index” (Wang et al. 2018; Zhang et al. 2020), the fuzzy logic method coupled with WQI (Moghari et al. 2015). For the irrigation uses, the evaluation of water quality is based on classic indices such as the electrical conductivity “EC,” the percent sodium “Na%,” alkalinity hazard “SAR,” and Kelly ratio “Kr.”

In Africa and specifically in Tunisia, which groundwater is practically the main water’s source in many regions, the evaluation of water quality was taking, recently, many attentions by the hydrogeologists which show that various regions are facing a decline in groundwater quality (Ghouili et al. 2018; Mnassri et al. 2018; Hamzaoui-Azaza et al. 2020).

The Hajeb Layoun-Jelma basin (HJB), which is the subject of this study, is located in central Tunisia. It is extending for over 1380 km^2^ which corresponds to 0.8% of the national territory and has about 172.003 inhabitants (INS 2014), which correspond to approximately 1.54% of the Tunisian population and which was 50,306 inhabitants in 1972 (Koschel 1980). The population growth (more than three times) plays a strong effect in the water request and has a big effect on water resources. The HJB aquifer system is of importance to the economic activity of both the southern and the central part of Tunisia. The water of the deep aquifer is transported to the Sfax city located at 180 km far away from the HJB. During the last decades, the HJB presented a development of agriculture activities, which is based on the uses of fertilizers and pesticides for improving agricultural production. This development has affected significantly pressure on groundwater resources: the water extraction increases for the both aquifers (shallow and deep aquifer) from 14.8 × 10^6^ in 1973 to 58.45 × 10^6^ m^3^ in 2018 with almost 2328 shallow wells and 137 deep wells (DGRE 1973–2018a). These human activities have putted increasing pressure on groundwater quality of theses aquifers.

In order to check the safety of HJB’s water, 28 water samples collected from shallow and deep aquifers tapping the HJB have been interpreted using statistical and geochemical methodologies to wholly understand the patterns of groundwater quality distribution. The principal aims of this research is to study the groundwater hydrochemistry and identify the purposes of water use of the HJB for either human consumption, irrigation using combined through GIS, or geochemical methods.

## Study area

### Site description

**Fig. 1:**
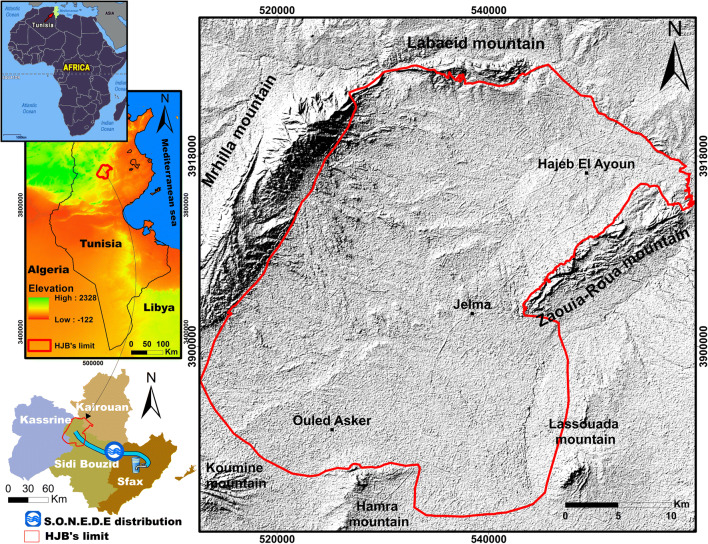
Location and elevation of the HJB

The Hajeb Layoun-Jelma basin located in the north-east central part of Tunisia. It is, approximately, located between *x* = 35° 00′ 00″, *y* = 8° 30′ 00″, and *x* = 35° 30′ 00″, *y* = 9° 00′ 00″ and extending for over 1380 km^2^. It comprises three regions (Sidi Bouzid, Kairouan, and Kassrine) with different occupied area; the maximum area of HJB is covered by the Sidi Bouzid region (Fig. 1). The HJB present a wide NE-SW directed syncline surrounded by various mountains; it is bordered to the north by the Labaeith mountain, to the south by the Hamra mountain, to the east by the Zaouia-Roua mountain, to the west by the Mrhilla mountain, to south-east by the Lessouda mountain, and to the south-west by the Koumine mountain (Fig. 1). The maximum altitude of HJB is 1384 m.

The HJB is characterized by semiarid climate, January presents the coldest month (mean temperature ≈ 11.8 °C), and the hottest is August (mean temperature ≈ 29.4 °C). The mean annual precipitation in Hajeb Layoun-Jelma basin, over the period 1972–2017, is 230 mm. The irrigation practices and the drinking supply for three regions (Sidi Bouzid, Kairouan, and Kassrine) are maintained by the water of HJB. The National Water Supply and Distribution Company (S.O.N.E.D.E) transport the water of the HJB to Sfax (Fig. 1), which is used for drinking purposes.

### Geology and hydrogeology

**Fig. 2:**
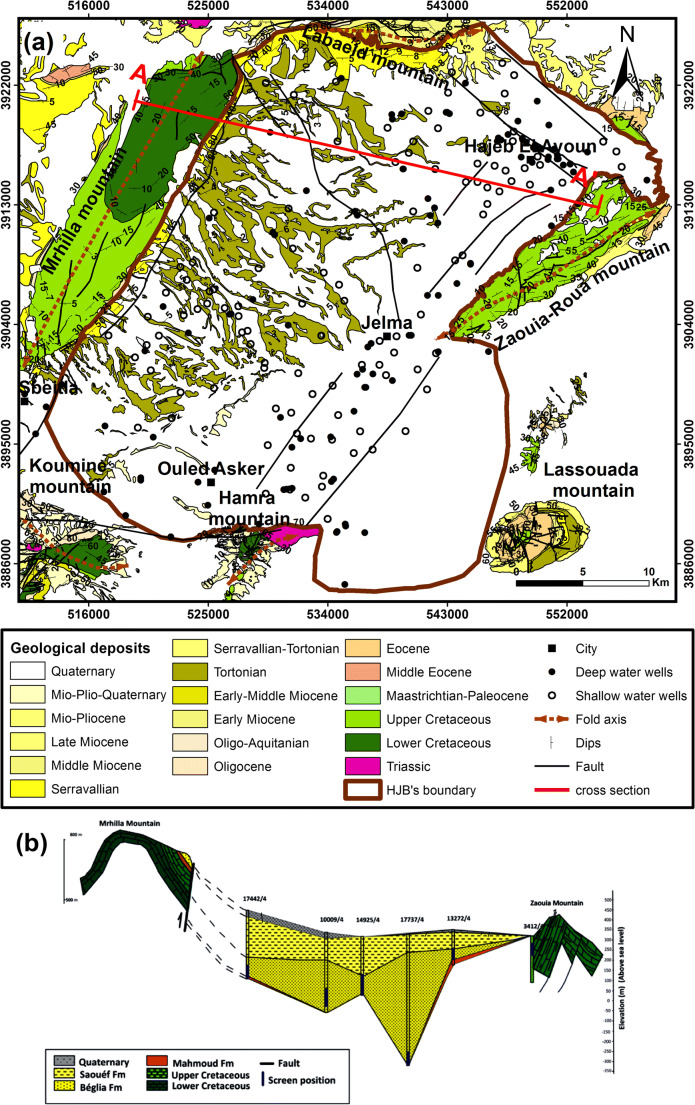
**a** Geologic map of HJB and **b** cross-section showing the principal formations in HJB (based on Jallalia et al. 2015)

The study area presents a geology series from Triassic to Quaternary with the missing of the Jurassic series (Fig. 2) (Koschel 1980; Jallalia et al. 2015; Thebti et al. 2018). The HJB is a collapse pan filled by Neogene and Quaternary deposits closed by anticlines (Fig. 2a).

The HJB is composed by multilayer aquifer system (Fig. 2b) (Jallalia et al. 2015; Thebti et al. 2018). The HJB is structured by various aquifer layers: the Cretaceous, the Miocene, and the Mio-Plio-Quaternary aquifers which coincide with the following local formations (from the bottom to the top): Abiod, El Gueria, Ain Grab, Beglia, Segui, and Quaternary deposits. The Beglia aquifer is usually confined, due to the superimposition of the clayey Saouaf formation. However, in HJB’s southern part, this aquiclude has been eroded, therefore allowing the Beglia formation to be closer to Mio-Plio-Quaternary aquifers, with an interposition of a lateritic layer (Koschel 1980). Due to the lateral discontinuity of the lateritic layer, somewhere, the Mio-Plio-Quaternary and the Beglia aquifers can interact from the hydraulic point of view (Koschel 1980).

### Abstraction and piezometry

The Hajeb Layoun-Jelma basin is composed by two mean aquifers (the most exploited aquifers); the shallow aquifers (Mio-Plio-Quaternary) and the first deep aquifer which coincide with the Beglia local formation.

**Fig. 3:**
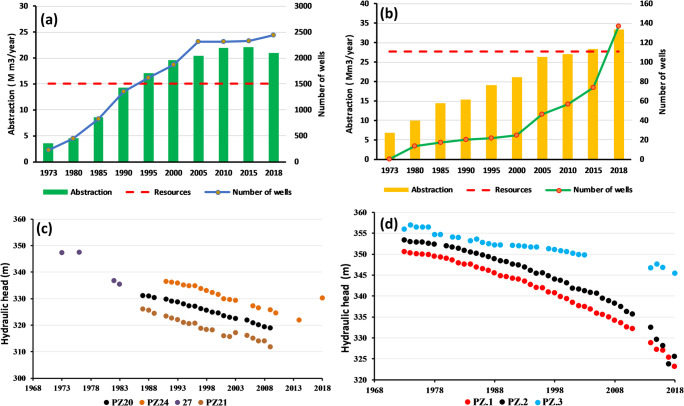
Evolution of groundwater abstraction and number of wells. **a** Shallow aquifer. **b** Deep aquifer and the decline of the piezometric level. **c** Shallow aquifer. **d** Deep aquifer

The HJB’s shallow aquifer is drilled by 2328 wells and the deep one is captured by 137 wells (DGRE 2018). The most of deep wells are located in Labaidh region, Ben Mrad region, and Felta and El Soud region. The total abstraction of HJB, in 2018, is equal to 58.45 × 10^6^ m^3^; however, the total renewable resources are equal to 42.8 × 10^6^ m^3^ which indicate a deficit of 15.65 × 10^6^ m^3^ (DGRE 2018). For the shallow aquifer, in 2018, the resources are calculated by DGRE equal to 15 × 10^6^ m^3^ and the abstraction equal to 20.94 × 10^6^ m^3^/year which indicate an abstraction of 140% with deficit equal to 5.94 × 10^6^ m^3^. This over-abstraction engendered the decrease of the water quality. In fact, in the last decades, the water salinity of the shallow aquifer was increased from 0.5 to 1 g/l (DGRE 2018). This over-exploitation is manifested by the increase of the number of wells (Fig. 3a): in 1974, 226 shallow wells tapped the shallow aquifer with an extraction rate equal to 7.94 × 10^6^ m^3^/year; in 2018, the number of wells increased to attend 2328 wells extracting a volume equal to 20.94 × 10^6^ m^3^/year (Fig. 3a) (DGRE 1974–2018).

The Beglia aquifer presents a good quality in many regions of HJB, which is transported, by S.O.N.E.D.E, to supply by drinking water the Sidi Bouzid and Sfax government. The S.O.N.E.D.E exploitation, of Beglia aquifer, exceeded 20 × 10^6^ m^3^/year (DGRE 2018). The total abstraction of this aquifer is equal to 33.4 × 10^6^ m^3^ in 2018 (Fig. 3b) which indicate an abstraction of 120% (resources equal to 27.8 × 10^6^ m^3^).

This over-exploitation of the both aquifers resulted in the decrease of the piezometric levels (Fig. 3c and d). For the shallow aquifer, the average yearly piezometric decline, over the period 1973–2018, equals to 0.4 m/year (DGRE 1973–2018b) (Fig. 3c). For the deep aquifer, from 1973 to 2018, the over-exploitation resulted in a high total decline of piezometric levels, average equal to 29.9 m (Fig. 3d) (DGRE 1973–2018b), which signify that this aquifer has a yearly piezometric decline equal to 0.7 m/year (Fig. 3d).

**Fig. 4:**
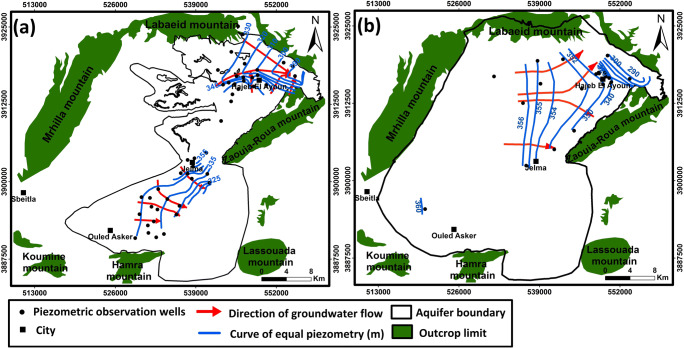
Piezometric maps. **a** Shallow and **b** deep aquifers

For the deep aquifer, the main groundwater flow direction is from the west coming from Mrhilla Mountain (Recharge zone), toward the central part of Hajeb Layoun where groundwater is divided in two parts: the first discharges at Hajeb Layoun fault and the second at the level of some faults in the north part of Zaouia-Roua Mountain (Fig. 4). The discharge areas are manifested by springs. For the shallow aquifer, the main flow direction is from the east to the west in the south part and two direction flows in the north part: east to the west and south to the north (Fig. 4).

### Land use

**Fig. 5:**
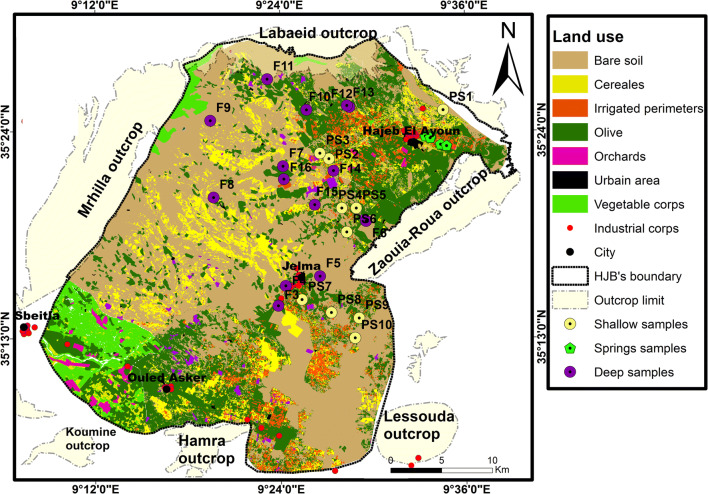
Land use map of HJB extracted from the agriculture map obtained from Regional Direction of Agriculture Development of Sidi Bouzid (CRDA-Sidi Bouzid)

The land use/land cover map of Hajeb Layoun-Jelma basin, published by DGRE in 2004, shows that the main type of agriculture is the irrigated and non-irrigated annual crops of olive (Fig. 5); these types of crops need high amounts of water with the use of huge quantities of fertilizers as well as to increase production, which influence on groundwater quality. Urban areas are also a potential source of pollution: in fact, the non-treated sewage rejected, by the ONAS (National Sanitation Office), in the natural environment of Hajeb Layoun-Jelma basin, which is estimated to an average of 400 m^3^ by day (DGRE 2017) can have a long-term influence on groundwater resources.

## Materials and methods

### Samples collection and analysis

In February 2017, a total of 28 samples were taken from wells in Hajeb Layoun-Jelma basin (humid period): 14 samples from the Beglia aquifer, 10 from the shallow aquifer (from depth of approximately 10–50 m), and 4 from springs (Fig. 5). In field, in order to avoid residual water’s influence, each well was pumped, for at least 30 min, until steady-state chemical conditions were obtained. According to the standard procedures given by Eaton (1950), the samples of HJB were collected using pre-cleaned and rinsed (distilled water and water sample) polyethylene bottles (1 L). The physical parameters (including temperature (T), pH, and electrical conductivity (EC)) were measured in the field (under minimal atmospheric contact) using handheld analyzing kits, which was calibrated first in the laboratory using standard solutions before use. After sampling, samples were labeled, taken to the laboratory, and stored below 4 °C. The chemical-analyzed parameters include major anions and cations (sodium (Na^+^), potassium (K^+^), calcium (Ca^2+^), magnesium (Mg^2+^), chloride (Cl^−^), bicarbonates (HCO_3_^−^), and sulfate (SO_4_^2−^)).

In order to validate the analysis results, the charge balance errors (%E) was calculated, for all samples, using the following formula:

1 $$ \%\boldsymbol{E}=\frac{\sum \mathbf{C}-\sum \mathbf{A}\ }{\sum \mathbf{C}+\sum \mathbf{A}} $$

where C is cations in meq/l and A is anions in meq/l.

The charge balance error checking of HJB’s samples showed that the results of analysis are judged perfectly (average %E ≈ 1.59% < 5%)

### Hydrochemical characterization

#### Conventional methods

The identification of hydrochemical processes, for the both aquifers of HJB (shallow and deep), was obtained by constructing several diagrams such as Piper diagram (Piper 1944) and Chadha diagram (Chadha 1999).

#### Origin of mineralization

Different reactions can be derived from the water-rock interaction, then defining the chemical water type. To understand the chemical processes, we have elaborated the correlation matrix, and also, we have established some correlations between selected major ions. These correlations can help to analyze the primary reactions that have formed current water chemistry and identify the origin of groundwater mineralization.

The Gibbs’ diagram (Gibbs 1970) was also used to understand the main mechanisms governing groundwater chemistry.

#### Multivariate statistical analysis

In the geochemical study, the separate study of each variable is an important phase in the analysis of chemical behavior, but it is often insufficient. Therefore, the data should be analyzed taking into account their multidimensional nature (Hamzaoui-Azaza et al. 2011).

Multivariate statistical analysis (MSA) is a multidimensional analysis widely used to identify the sources of solutes in a groundwater system and to well understanding of water quality. It allows the comparison of all samples of water and the identification of their different solutes’ origin (Hamzaoui-Azaza et al. 2011). MSA was chosen to determine the inter-data relationships of the HJB’s samples. In total, 12 physico-chemical parameters were analyzed in 28 samples collected in 2017; these variables (pH, EC, salinity, O_2_, Na^+^, Ca^2+^, Mg^2+^, K^+^, Cl^−^, HCO_3_^−^, and SO_4_^2−^) were successfully used in principal component analysis. The parameters used in MSA referred to different units of measurement (meq/l, us/cm…), so their values should be standardized; we have used the following transformation function (Medina-Gomez and Herrera-Silveira 2003):

2 $$ Z=\left(X-\upmu \right)/\upsigma $$

where *Z* is the standardized value, *X* the original value of the measured parameter, μ the mean of the variable, and σ the standard deviation.

## Water quality assessment

### Drinking use

#### Standards of drinking

In order to maintain the human health, the World Health Organization (WHO) has set limit values not to be exceeded if want to respect international standards of consumption. Also, all countries, of the world, do not follow the same standards; each country has defined their propriety standards of drinking water quality, some adopt their own standards, and others choose those recommended by the WHO (2011). Tunisia has fixed national standards (NT.09.14) for the potability of the water. The difference between the Tunisian standards and WHO limits reflects the required management of water in Tunisia.

### Drinking index

The assessment of suitability for drinking purpose, in HJB, was evaluated using three indices: water quality index (WQI), entropy water quality index (EWQI), and improved water quality index (ImpWQI).

#### Water quality index

The WQI method is frequently used to assess the drinking water’s quality (Ghouili et al. 2018; Asadi et al. 2020).

The calculation of WQI is based on the standards suggested for uses, where 9 groundwater quality parameters are considered: pH, EC, HCO^3−^, Cl^−^, SO_4_^2−^, Ca^2+^, Mg^2+^, Na^+^, and K^+^. For computing the WQI, weights (*wi*) are assigned for each parameter: the weight of “5” has been attributed to five parameters: EC, Mg^2+^, Na^+^, Cl^−^, and SO_4_^2−^ due to their major role in quality assessment. A minimum weight equal to “1” has been given to HCO_3_^–^ and k^+^ since their less significant role in quality evaluation and medium weights of 2 and 3 has been assigned to Ca^2+^ and pH.

The WQI is computing on following up the Eqs. (3), (4), and (5):

3 $$ RWi=\frac{wi}{\sum_{i=1}^n wi} $$

4 $$ Qi=\frac{Ci}{Si}\times 100 $$

5 $$ \mathrm{WQI}=\sum RWi\times Qi $$

where *wi* is the weight for each parameter, *RWi* relative weight for each parameter, *n* number of parameters, *Ci* concentration of parameter *i* (each water sample, (mg/L)), and *Si* drinking use’s standard (WHO 2011).

The ranges of water quality were determined according to the WQI; we have classified the water samples according the ranges of WQI values (Table 1). Spatial distribution of WQI values were prepared using a weighted inverse-distance interpolation (IDW) technique.

**Table 1:** Classification of groundwater quality based on WQI, EWQI, and ImpWQI

Index	< 50	50–100	100–150	150–200	> 200
Rank	1	2	3	4	5
Water quality	Excellent	Good	Medium	Poor	Extremely poor

#### Entropy water quality index

The EWQI is widely applied to assess the drinking water’s quality (Wu et al. 2011; Islam et al. 2017).

For computing the EWQI, according to Islam et al. (2017), when *m* water samples (i = 1, 2,…, *m*) are taken to evaluate the quality and each sample is analyzed for “n” parameters (j = 1, 2,…, *n*), the following steps have been followed:

In the first step, eigenvalue matrix, A, was constructed as follows:

6 $$ \mathrm{A}=\left|\begin{array}{cccccc}\mathrm{A}11& \mathrm{A}12& .& .& .& \mathrm{A}1\mathrm{n}\\ {}\mathrm{A}21& \mathrm{A}22& .& .& .& \mathrm{A}2\mathrm{n}\\ {}A31.& A32.& .& .& .& A3n.\\ {}.& .& .& .& .& .\\ {}.& .& .& .& .& .\\ {}\mathrm{A}\mathrm{m}1& \mathrm{A}\mathrm{m}2& .& .& .& \mathrm{A}\mathrm{m}\mathrm{n}\end{array}\right| $$

After, matrix A is converted into a standard-grade matrix B (Eq. (8)) using Eq. (7).

7 $$ \left\{\begin{array}{c}\mathrm{Bij}=\frac{Aij- Aij\ \min }{Aij\ \max - Aij\ \min}\kern0.75em \mathrm{for}\ \mathrm{efficiency}\ \mathrm{type}\ \mathrm{paramaters}\\ {}\kern0.5em \\ {}\mathrm{Bij}=\frac{Aij\ \max - Aij\ }{Aij\ \max - Aij\ \min}\kern3.25em \mathrm{for}\kern0.5em \mathrm{cost}\ \mathrm{type}\ \mathrm{paramaters}\end{array}\right. $$

8 $$ \mathrm{B}=\left|\begin{array}{cccccc}\mathrm{B}11& \mathrm{B}12& .& .& .& \mathrm{B}1\mathrm{n}\\ {}\mathrm{B}21& \mathrm{B}22& .& .& .& \mathrm{B}2\mathrm{n}\\ {}B31.& B32.& .& .& .& B3n.\\ {}.& .& .& .& .& .\\ {}.& .& .& .& .& .\\ {}\mathrm{B}\mathrm{m}1& \mathrm{B}\mathrm{m}2& .& .& .& \mathrm{B}\mathrm{m}\mathrm{n}\end{array}\right| $$

Then, the entropy weight (Wj), for each parameter, is calculated as follows:

9 $$ \mathrm{Wj}=\frac{1- ej}{\sum_{i=1}^m\left(1- ej\right)} $$

where

10 $$ \mathrm{ej}=\frac{1}{Ln\ m}{\sum}_{i=1}^m Pij\ln (Pij) $$

and

11 $$ \mathrm{Pij}=\frac{1+ Bij}{\sum_{i=1}^m\left(1+ Bij\right)} $$

The rating quality is calculated for the *n* parameters (j = 1, 2 …., *n*) for all the samples, using the concentration of parameter j (Cj) and the standard limit (Sj), using the following formula:

12 $$ \mathrm{qj}=\frac{Cj}{Sj}\times 100 $$

In this study, the rating quality is calculated based on the WHO standard ( 2011).

Finally, the EWQI is calculated as follows:

13 $$ \mathrm{EWQI}={\sum}_{j=1}^m Wj\times qj $$

#### Improved water quality index

The ImpWQI is widely used for assessing the drinking water quality (Zhang et al. 2020). For computing the ImpWQI, the first step is to determinate the weights of the different used parameters. Firstly, the data was normalized to eliminate the units’ influence. To calculate the weight of parameters, the CRITIC weighting (Zhang et al. 2020) was used (Eq. (14)–(16)).

The ImpWQI, for each sample, are calculated on following up these equations:

14 $$ Cij=\frac{\sum \left(\mathrm{aij}-\overline{\mathrm{aij}}\right)\left(\mathrm{bij}-\overline{\mathrm{bij}}\right)}{\sqrt{\sum {\left(\mathrm{aij}-\overline{\mathrm{aij}}\right)}^2\times \sum {\left(\mathrm{bij}-\overline{\mathrm{bij}}\right)}^2\kern0.75em }} $$

15 $$ Fj=\hbox{\pounds}j\ {\sum}_{j=1}^m\left(1- cij\right) $$

16 $$ Wj= Fj/{\sum}_{j=1}^m Fj $$

17 $$ qj=\frac{\mathrm{aij}}{Sj}\times 100 $$

18 $$ \mathrm{ImpWQI}={\sum}_{j=1}^m Wj\times qj $$

where a_ij_ and b_ij_ are the original and the normalized data value, respectively, $\overline{\mathrm{aij}}$ and $\overline{\mathrm{bij}}$ the average of a_ij_ and b_ij_, respectively, *Fj* the information amount of the *j*th parameter, £j standard deviation of the *j*th parameter, *c* correlation coefficient, *m* total number of parameter, and *Wj* the weight of the *j*th parameter. *qj* is the rating of the *j*th parameter and *Sj* the standard limit of the *j*th parameter (WHO 2011).

The obtained results from the three drinking indices were classified into five classes (Table 1).

##### Irrigation suitability assessment

Different ionic parameters (in meq/l) were used to assess the irrigation water quality in HJB basing on various indices such as TH (total hardness) (Todd 1980), EC (electrical conductivity (μs/cm)), SAR (alkalinity hazard) (Richard 1954), Na% ( percent sodium) (Wilcox 1955), MH (magnesium hazard) (Raghunath 1987), KR (Kelley ratio) (Kelly 1951), and PI (permeability index) (Doneen 1964) (Eqs. (19)–(24)):

19 $$ \mathrm{TH}=2.5\times \mathrm{Ca}+4.1\times \mathrm{Mg} $$

22 $$ PI=100\times \frac{\mathrm{Na}+\sqrt{\mathrm{HCO}3}}{\mathrm{Na}+\mathrm{Mg}+\mathrm{Ca}} $$

24 $$ Mh=\frac{\mathrm{Mg}}{\mathrm{Ca}+\mathrm{Mg}} $$

## GIS analysis

A GIS database was developed to make useful tools from available data to greater understand the functioning of HJB. Under ArcGis 10.3, a database has been established including the inventory of all deep and shallow wells implemented in different aquifers and their main characteristics (localization, year of creation, borehole depth) and historical data (rainfall, piezometry, and withdrawals). The thematic maps, such as piezometric maps, geological maps, land use, and distribution maps of some parameters such as salinity and quality indices of study area, were obtained from 1:50000 scale and were georeferenced under the UTM coordinate system. The coordinate of each well was measured by using, in the field, a global positioning system (GPS). The spatial distribution of different indices such as salinity, WQI, EWQI, and ImpWQI were obtained by the IDW method.

## Results and discussion

**Fig. 6:**
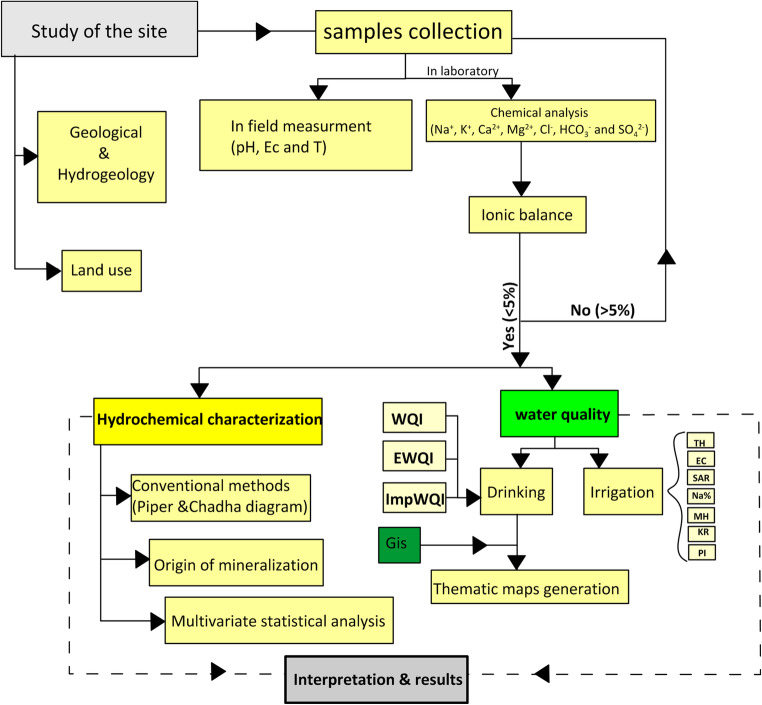
Flow chart showing the methodology applied in the HJB’s water evaluation

The steps followed, in this research, are resumed in Fig. 6.

### Hydrochemical data

A statistical view of hydrochemical parameters (min, max, and standard deviation) is given in Table 2. The pH data ranged from 7.15 to 8.45 and 7.63 to 8.24 for the shallow and the deep samples, respectively. These results show that the both aquifers have a pH close to neutrality with a slight tendency toward the basic composition. The temperatures are characterized by heterogeneous values varying from 10.3 to 24.8 °C and 13.1 to 30.2 °C for the shallow and the deep samples, respectively. The temperature of water depends on the well depth, with an average value and standard deviation equal to 17.9 °C and 3.97 °C, for the shallow and springs samples, and equal to 22.8 °C and 4.96 °C for the deep samples. For the shallow and springs samples, the electrical conductivity values vary from 1544 to 9770 μs/cm with a mean of 2685 μs/cm. For the deep samples, the EC varies from 393 to 3960 μs/cm with a mean of 1729 μs/cm.

**Table 2:** Statistical summary of the physical and chemical parameters of HJB samples (ionic contents in mg/l)

		T (°C)	PH	EC	Salinity	Na^+^	Ca^2+^	Mg^2+^	K^+^	Cl^−^	HCO^3−^	SO_4_^2−^
Deep	Min	13.10	7.15	393	0.10	17.48	5.80	0.47	2.34	82.36	32.33	4.80
	Max	30.20	8.45	3960	1.80	459.31	55.60	117.67	15.60	935.43	154.33	105.12
	SD	4.96	0.35	961.82	0.52	117.60	13.09	29.16	3.89	240.49	37.09	32.80
Shallow /springs	Min	10.30	7.63	1544	0.70	142.60	37.80	41.80	4.68	341.16	32.33	1.44
	Max	24.80	8.24	9770	6.50	1075.02	70.40	148.23	19.89	1768.61	305.00	235.20
	SD	3.97	0.20	2014.12	1.41	230.09	8.38	27.55	4.71	368.03	87.15	65.05

For the both type of samples (shallow/springs and deep), the chemical analysis indicated that the abundance order of the major cations is Na > Mg > Ca > K. For the shallow and springs samples, the concentration of major cations, Na^+^, Ca^2+^, Mg^2+^, and K^+^, are ranged from 142.6 to 1075, 37.8 to 70.4, 41.8 to 148.23, and 4.68 to 19.89 mg/l with a mean value of 265.54, 47.2, 84.38, and 7.61 mg/l, respectively. For the deep samples, the cations, Na^+^, Ca^2+^, Mg^2+^, and K^+^, are ranged from 17.48 to 459.31, 5.8 to 55.6, 0.47 to 117.67, and 2.34 to 15.6 mg/l with a mean value of 138.35, 37.7, 35.53, and 4.88 mg/l, respectively. The order of abundance of anion is Cl > HCO_3_^−^ > SO_4_. The abundance of these cations and anion is derived from a mineralization process, which can be natural or anthropogenic.

**Fig. 7:**
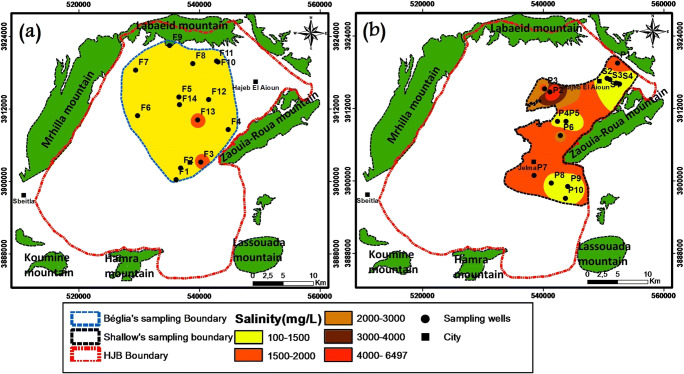
Spatial distribution of salinity. **a** Deep and **b** shallow aquifer. The map was plotted using the IDW method

The groundwater salinity shows a wide variation from 100 to 1800 mg/l with a mean value equal to 700 mg/l and from 700 to 6500 mg/l with a mean value equal to 1400 mg/l for the deep and the shallow aquifers, respectively. The distribution of the salinity presented in Fig. 7 reveals that in the shallow, aquifer has high soluble salts in the totality of samples (one sample, salinity < 1 and 13 samples, salinity > 1 g l^−1^ with one sample exceeding 6 g l^−1^) (Fig. 7). The deep aquifer has moderate salinity: 3 samples exceeding 1 g l^−1^ and the rest (11 samples) indicate salinity less than 1 g l^−1^. The high salinity values would be related to the leaching of salts from soils, the use of fertilizers in agriculture activities, or/and return flow from irrigation water (Mnassri et al. 2018). This hypothesis is confirmed by analyzing the samples that are taken from wells located in the irrigated perimeters (see Fig. 5).

### Groundwater mineralization processes

#### Correlation of parameters

The correlation matrix of the shallow and springs samples indicated that the contents of sodium, magnesium, chloride, and calcium are high positively correlated with salinity (Table 3(a)). These positive correlations indicate the continuous addition of these ions along groundwater flow path. Therefore, these elements contribute to the groundwater mineralization. The concentration of Cl^−^ is correlated with Na^+^ with a correlation index of 0.95, indicating that the halite dissolution may be the important reaction affecting the water chemistry. The electrical conductivity also shows a perfect positive correlation with Na^+^ (*R* = 0.98), Ca^2+^ (*R* = 0.82), salinity (*R* = 0.98), Cl^−^ (*R* = 0.95), and moderately positive correlation with Mg^2+^ (*R* = 0.67).

**Table 3:** Pearson correlation matrix of HJB. (a) Shallow wells/springs, and (b) deep wells. Italics indicates significant 50% confidence level

	T (°C)	PH	EC	Salinity	Na^+^	Ca^2+^	Mg^2+^	K^+^	Cl^−^	HCO_3_^−^	SO_4_^2−^
(a)											
T (°C)	1										
PH	− 0.53	1									
EC	0.08	− 0.26	1								
Salinity	0.03	− 0.24	*0.98*	1							
Na^+^	0.12	− 0.33	*0.98*	*0.98*	1						
Ca^2+^	− 0.34	− 0.05	*0.82*	*0.82*	*0.77*	1					
Mg^2+^	− 0.02	− 0.11	*0.65*	*0.63*	*0.63*	*0.72*	1				
K^+^	− 0.05	− 0.28	− 0.22	− 0.14	− 0.16	− 0.08	0.09	1			
Cl^−^	0.02	− 0.26	*0.95*	*0.95*	*0.95*	*0.86*	*0.69*	− 0.11	1		
HCO_3_^−^	0.25	0	0.20	0.20	0.21	− 0.04	0.34	0.13	0.03	1	
SO_4_^2−^	0.07	− 0.10	0.17	0.12	0.16	0.01	0.12	− 0.19	− 0.05	0.47	1
(b)											
T (°C)	1										
PH	*− 0.53*	1									
C25°C	0.47	*− 0.52*	1								
Salinity	0.39	− 0.55	*0.98*	1							
Na+	0.49	− 0.49	*0.97*	*0.95*	1						
Ca2+	0.26	*− 0.65*	*0.77*	*0.82*	*0.70*	1					
Mg2+	0.14	− 0.36	*0.75*	*0.80*	*0.70*	*0.69*	1				
K+	*0.59*	− 0.21	*0.72*	*0.65*	*0.77*	0.38	0.40	1			
Cl−	0.40	− 0.40	*0.96*	*0.95*	*0.97*	*0.71*	*0.80*	*0.75*	1		
HCO3−	0.42	− 0.57	*0.61*	*0.58*	*0.62*	*0.61*	0.40	*0.56*	*0.58*	1	
SO42−	0.07	− 0.30	0.37	0.46	0.32	0.45	*0.60*	− 0.02	0.34	− 0.08	1

The matrix of the deep samples (Table 3(b)) indicates that EC shows a high correlation (positive) with salinity (*R* = 0.98), Na^+^ (*R* = 0.97), and Cl^−^ (*R* = 0.96) and moderately positive correlation with Ca^2+^, Mg^+^, K^+^, and HCO_3_^−^ with correlation value equal to 0.77, 0.75, 0.72, and 0.61, respectively. Na^+^ also shows a high correlation index (positive) with all the major ions except SO_4_^2−^. The high correlation observed between some parameters suggests the extent of interdependence and also suggests that these ions may be derived from a common source.

### Identification of water-rock interaction

**Fig. 8:**
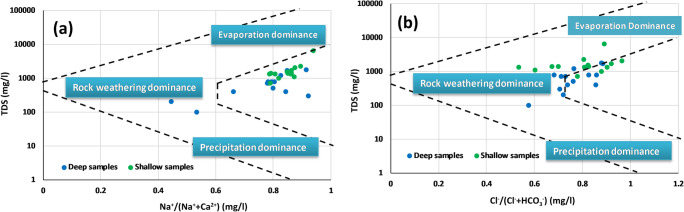
Gibbs’ diagrams of the shallow and deep aquifers of HJB. **a** Ratio I vs. TDS and **b** ratio II vs. TDS

To understand the main mechanisms governing groundwater chemistry, Gibbs’ diagrams have been used. The weight ratios of ratio I: (Na^+^/(Na^+^+Ca^2+^)) and ratio II: (Cl^−^/(Cl^−^ + HCO_3_^−^)) are plotting as a function of total dissolved solids (TDS), representing Gibbs’ diagrams. This diagram is used to identify the origin of dissolved constituents, such as rock weathering dominance, precipitation dominance, and evaporation dominance or by combination of these influences (Gibbs 1970). According to the Gibbs’ diagrams (Fig. 8), the data indicates that the chemical composition’s HJB samples are governed by evaporation and rock weathering. The importance of evaporation processes and rock weathering are also confirmed by the calculation of Hounslow ratio (Cl^−^/Σ anions) which indicates, for the both aquifers, two chemical sources: evaporate or brine water sources (ratios > 0.8 and TDS > 500) and rock weathering (ratios < 0.8) (Hounslow 1995).

**Fig. 9:**
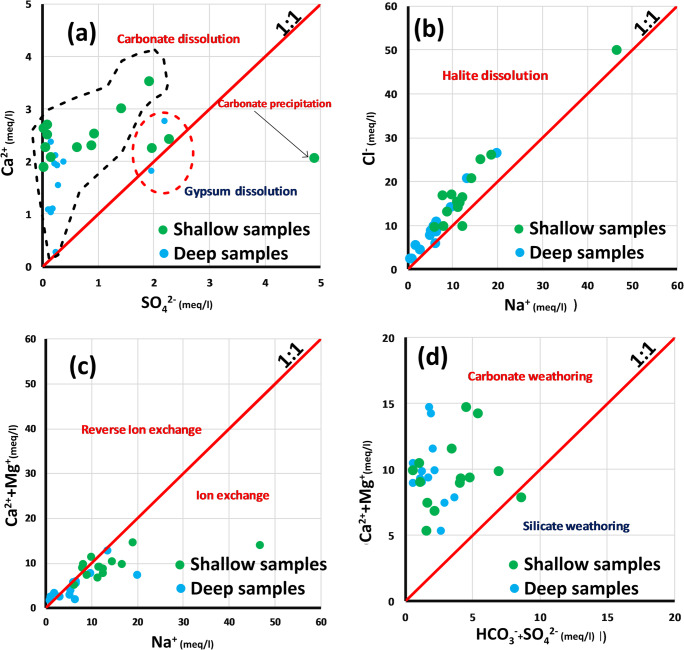
**a** Plot of SO_4_^2−^ against Ca^2+^. **b** Plot of Na^+^ against Cl^−^. **c** Plot of Na^+^ against (Ca^+^+Mg^+^). **d** Plot of (HCO^−^_3_ + SO_4_^2−^) against (Ca^2+^+Mg^2+^) in meq/l in shallow and deep aquifer water samples.

A plot of Ca^2+^ and SO_4_^2−^ shows that for the shallow samples (Fig. 9a), one sample below the line 1:1 (PS 3) indicates a deficit in Ca^2+^, suggesting carbonate precipitation; two samples (PS10 and S1) are close to the bisector line (1:1), indicating that gypsum is the source of calcium, while the majority of samples are located above the dissolution straight line and indicated an excess in Ca^2+^, suggesting carbonate dissolution (Fig. 9a). For the deep samples, two samples (F11 and F14) are close to the bisector line (1:1), indicating that gypsum is a source of calcium, while the majority of the water samples are located above the dissolution straight line and indicated an excess in Ca^2+^, suggesting carbonate dissolution (Fig. 9a).

Evaporation process is also a major process in controlling the groundwater’s chemistry. The both type of samples (shallow/springs and deep) represented in Fig. 9b are very close to the bisector line (1:1) of sodium against chloride’s plot, suggesting that in these wells, salinity is controlled by halite dissolution.

According to scatter diagrams (Fig. 9c), the groundwater mineralization is controlled, in addition to minerals dissolution, by ion exchange with clay minerals present in the aquifers and also reverse ion exchange.

The indicator of carbonate and silicate weathering is confirmed by the (Ca^2+^+Mg^2+^) against (HCO_3_^−^ + SO_4_^2−^) scatter diagrams in Fig. 9d showing that:

- The shallow and springs samples are distributed at the left and the right part of the 1:1 (line). One sample indicating the abundance of SO_4_^2−^ + HCO_3_^−^ by 54% over Ca^2+^+Mg^2+^ is a sign of silicate weathering. The most of samples located in the left part of the 1:1 (line) indicates that the water samples are related to carbonate rock.
- The deep samples are distributed at the left part of the 1:1 (line) indicating a weathering of carbonates which represents the main source of bicarbonate ion.

### Hydrochemical water type

**Fig. 10:**
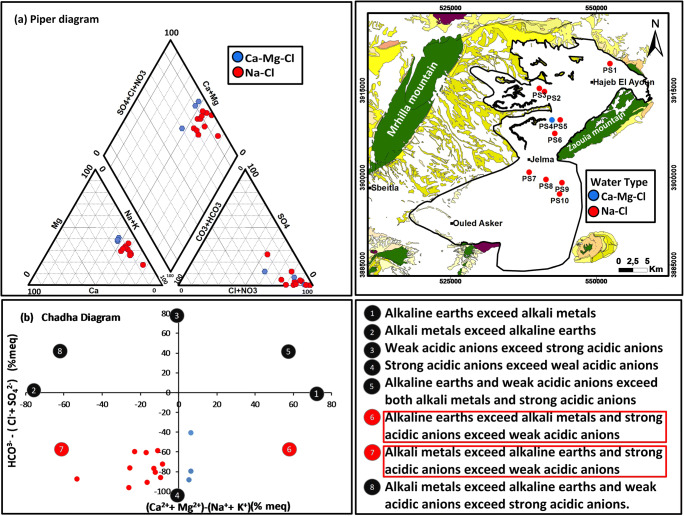
**a** Piper diagram and **b** Chadha diagram of the shallow samples

**Fig. 11:**
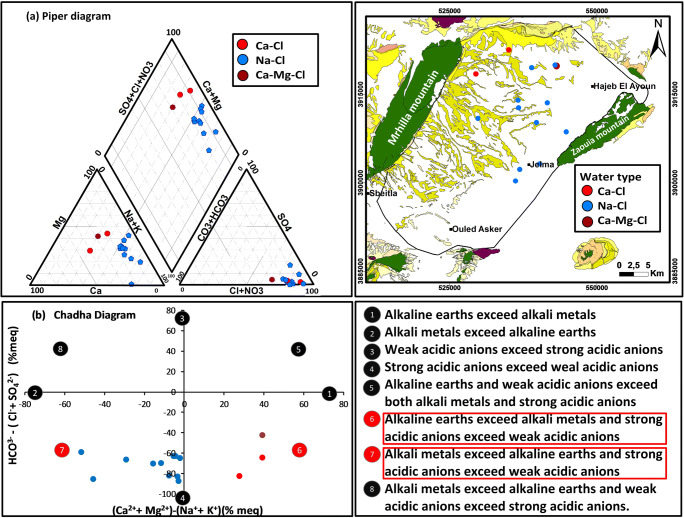
**a** Piper diagram and **b** Chadha diagram of the deep samples

Considering the piper trilinear plot (Figs. 10 and 11), we can distinguish three major groundwater groups for the deep aquifer: Na-Cl, Ca-Mg-Cl, and Ca-Cl and two water type for the shallow aquifer: Na-Cl and Ca-Mg-Cl. For the deep aquifer; the first group (Ca-Cl) type waters are highly mineralized. They represent the northwest part of Beglia aquifer (recharge zone). The high Ca^+^ concentration in the northwest part of Beglia aquifer is derived from dissolution of carbonate present in the cretaceous of Dj Mghilla. The second water type is Na-Cl; it presents 78% of samples for the deep aquifer and also for the shallow aquifer. The Na cation is derived from the ion exchange with the clay of the adjacent layer (Saouaf formation). Two much closed wells, in the deep aquifer, present two different water type (Na-Cl and Ca-Mg-Cl); the Na-Cl water type presents 78% of samples while Ca-Mg-Cl is present only in one sample. Based on the screen position of wells, we can detect that the well corresponding to the Ca-Mg-Cl water type presents very different screen position; so, we can conclude that Beglia aquifer presents vertical water-type stratification.

The chemical data of shallow/springs and deep samples, collected from the studied area, are plotted in the Chadha diagram presented in Figs. 10 and 11. All the samples fall in fields 6 and 7, and this means that “alkaline earths exceed alkali metals and strong acidic anions exceed weak acidic anion” and “Alkali metals exceed alkaline earths and strong acidic anions exceed weak acidic anions.”

### Multivariate statistical analyses

Principal component analysis was achieved for the two aquifers separately: a dataset of 28 samples (14 deep samples and 14 shallow and springs samples) and 12 physico-chemical elements to determine relationships between major elements and also physical parameters. Table 4 shows the eigenvalues, the percentage of variance, associated with each other, and the cumulative percentage.

**Table 4:** Variance explained by the first three principal components

Component		Eigenvalues	% total variance	% cumulative
Shallow samples	1	5.34	48.55	48.55
	2	1.92	17.45	66
	3	1.34	12.25	78.25
Deep samples	1	6.94	63.11	63.11
	2	1.39	12.64	75.76
	3	1.19	10.85	86.61

**Fig. 12:**
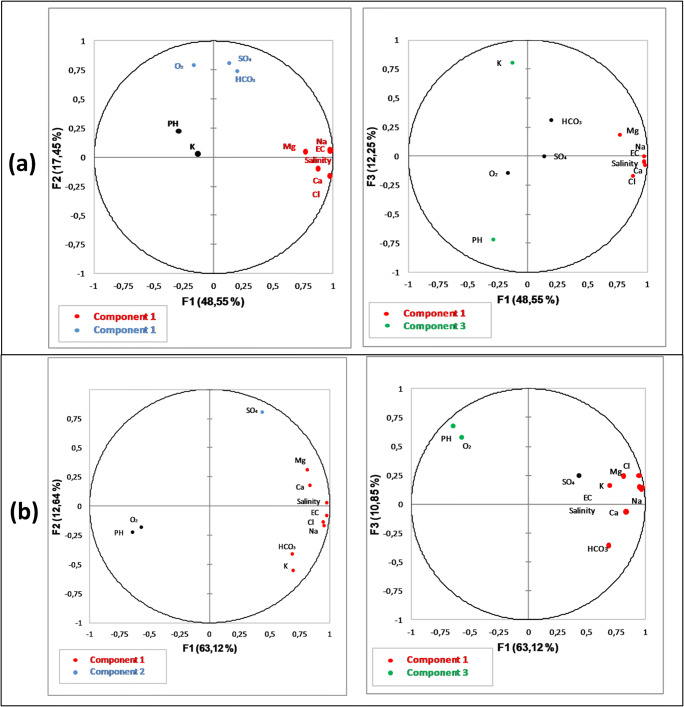
Projection of the variables in the first, second, and third factorial plan (principal component analysis) (**a**) including all shallow and springs samples in HJB and (**b**) deep samples in HJB

The results of the analysis presented in Fig. 12 reveal that the first three factors illustrate approximately 78%, of total variance, for the shallow and springs samples and 86% for the deep samples. For the shallow and springs samples, the first factor is responsible for about 48%, of total variance, and is well represented by salinity, Na^+^, EC, Mg, Ca^2+^, and Cl^−^. These elements ensure the mineralization of the shallow aquifer’s water. Consequently, component “1” is defined as the salinity component representing the weathering of halite and evaporate minerals. Component “2” is represented by O_2_, SO_4_^2−^, and HCO_3_^−^. Additional 12.25%, of total variance, was explained in F3 and was represented by K^+^, O_2_, and pH.

For the deep samples, the first factor is responsible for about 63.11%, of total variance, and is well represented by Mg^2+^, salinity, Na^+^, K^+^, Ca^2+^, HCO_3_^−^, Cl^−^, and EC; this component is defined as the salinity component representing the weathering of halite and evaporate minerals. Component 2 is represented by SO_4_^2−^ defined as a factor of sulfates. The third component represents 10.85%, of total variance, was explained in F3, and was represented by O_2_ and pH.

### Water quality

#### Drinking use

##### Standard limits

**Fig. 13:**
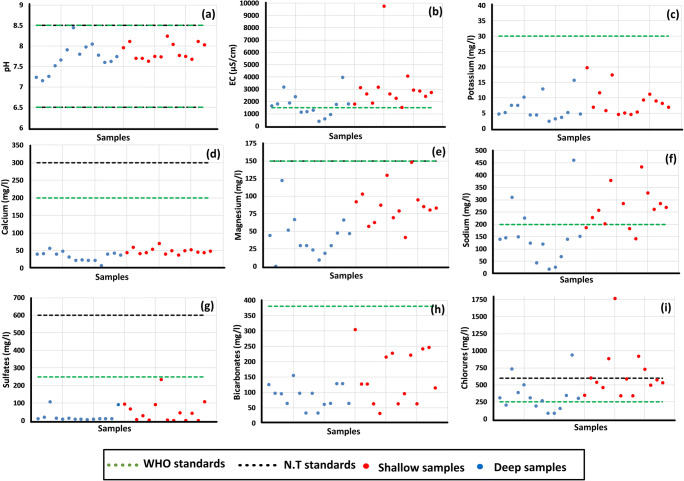
Comparison of major ion concentration (in mg/l) and physical parameters in HJB with the WHO standards and Tunisian norms (NT 09–14). **a** pH, **b** EC (μs/cm), **c** K^+^, **d** Ca^2+^, **e** Mg^2+^, **f** Na^+^, **g** SO_4_^2−^, **h** HCO_3_^−^, and **i** Cl^−^

The physical (pH and EC (μs/cm)) and chemical parameters (K^+^, Ca^+^, Mg^+^, Cl^−^, SO_2_^−^, Na^+^, HCO_3_^−^/in mg/l) were compared with the world’s standard (WHO 2011) and the national standard (NT 2013). As show in Fig. 13, all samples (*n* = 28) respect the maximum permissible limit, for the both WHO and NT standards, for the pH, the potassium (K^+^), the calcium (Ca^+^), the magnesium (Mg^+^), the bicarbonates (HCO_3_^−^), and the sulfates (SO_2_^−^). For the electrical conductivity (EC), the limit given by the WHO (1500 μs/cm) is not respected by all the shallow samples and the most of deep samples (58%). For the chlorides (Cl^−^), all the shallow samples exceeded the WHO limit (250 mg/l) and 29% of the shallow samples exceeded the national limit (600 mg/l). For the deep aquifer, 9 samples (64%) exceeded the WHO limit and two samples (14%) exceeded the national limit (600 mg/l). For the sodium (Na^+^) parameter, the permissible value given by the WHO (200 mg/l) was respected only by four samples (29%) in the shallow aquifer and exceeded by three samples (21%) from the deep one. In all collected samples, only one physical parameter and two major ions (one cation and one anion) not respect the WHO and NT limit in the most of samples. In the total, only 15% of samples respect the permissible limits, of all physico-chemical parameter, given by the WHO, which can affect the human health.

### Water quality indices

The evaluation of water quality, of HJB, for drinking uses was effectuated using three quality indices: EWQI, WQI, and ImpWQI.

The WHO standards were selected to calculate the quality rating scale (Q). The WQI ranged from 64.41 to 328.64 for the shallow aquifer and from 22 to 155.61 for the deep aquifer. It shows four classes of both aquifers (Table 5), extended from “good” to “extremely poor” for the shallow aquifer and from “excellent” to “poor” for the deep one. For the EWQI, the index value ranged from 55.29 to 248.41 for the shallow aquifer and from 22 to 122.8 for the deep aquifer. It shows three classes of both aquifers (Table 5, extended from “good” to “extremely poor” for the shallow aquifer and from “excellent” to “Medium” for the deep one. For the ImpWQI, the value ranged from 178.69 to 1011 for the shallow aquifer and from 43.93 to 475.6 for the deep aquifer. It shows various classes of both aquifers (Table 5), extended from “poor” to “extremely poor” for the shallow aquifer and from “excellent” to “poor” for the deep one.

**Table 5:** Classification of shallow and deep samples quality based on EWQI, WQI, and ImpWQI

Index		< 50	50–100	100–150	150–200	> 200
Water quality		excellent	Good	Medium	Poor	Extremely poor
EWQI	% shallow aquifer	-	79%	14%	-	7%
	% deep aquifer	50%	36%	14%	-	-
WQI	% shallow aquifer	-	36%	50%	7%	7%
	% deep aquifer	29%	57%	7%	7%	-
ImpWQI	% shallow aquifer	-	-	-	7%	93%
	% deep aquifer	7%	14%	22%	-	57%

A correlation was effectuated between the physico-chemical parameters, used in the calculation of the indices, and the three indices (Table 6). For the both aquifers, the three indices (ImpWQI, EWQI, and WQI) present a negative low correlation with the pH, a low correlation with sulfates (SO_4_^−^), and a strong correlation with the major physico-chemical parameters (EC, Na^+^, Ca^2+^, Mg^2+^, Cl^−^, HCO_3_^−^) except in the shallow aquifer the potassium K^+^ and the bicarbonates HCO_3_^−^ present a low correlation value with the three indices (Table 6). The correlation values are related to the parameter’s weight which is given in WQI method and calculated in the two indices (ImpWQI and EWQI). For the both types of samples (shallow/deep), the three indices indicate very similar correlation values but the EWQI indicate the high values with very negligible differences with the two other indices.

**Table 6:** Correlation between the various water quality indices (ImpWQI, EWQI, and WQI) and physico-chemical parameters for the deep and shallow aquifer

	Index	pH	EC	Na^+^	Ca^2+^	Mg^2+^	K^+^	Cl^−^	HCO^3−^	SO_4_^2−^
Deep aquifer	WQI	− 0.47	0.99	0.98	0.76	0.82	0.73	0.99	0.59	0.42
	EWQI	− 0.47	0.99	0.98	0.76	0.81	0.75	0.99	0.62	0.40
	ImprWQI	− 0.46	0.99	0.98	0.76	0.81	0.75	0.99	0.62	0.40
Shallow aquifer	WQI	− 0.29	0.99	0.99	0.84	0.7	− 0.16	0.97	0.2	0.16
	EWQI	− 0.33	0.99	0.99	0.83	0.72	− 0.12	0.97	0.24	0.17
	ImprWQI	− 0.27	0.99	0.98	0.85	0.69	− 0.16	0.99	0.13	0.07

**Fig. 14:**
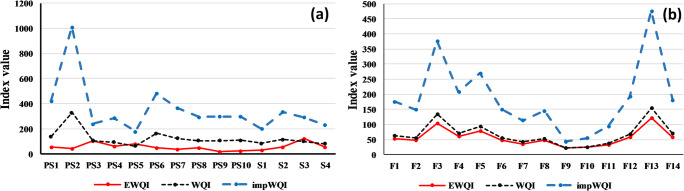
Comparison of the results of the WQI, ImpWQI, and EWQI indices using the WHO standard in (**a**) shallow aquifer and (**b**) Deep aquifer

Figure 14 shows the water quality index values calculated by the three proposed indices (WQI, ImpWQI, and EWQI) in the deep and shallow aquifers. The indices showed similar results, regarding EWQI and WQI. The ImpWQI indicate the higher index values; for the shallow samples, the ImpWQI indices are ranged from 178.69 to 1011 which indicate poor to extremely poor water quality. For the deep samples, the ImpWQI indicated that the samples with Na-Cl water type indicate the low water quality then the other water types.

**Fig. 15:**
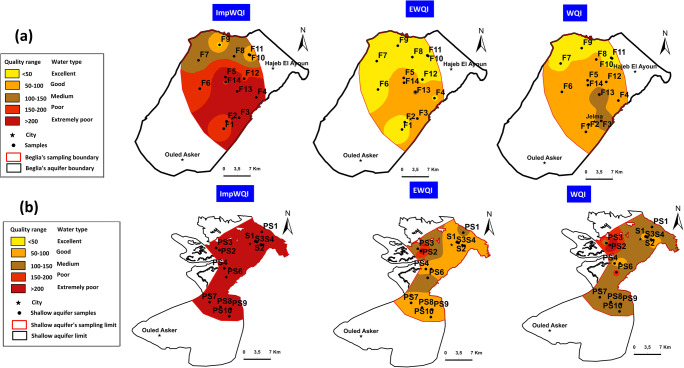
Distribution of the three indices (ImpWQI, EWQI, and WQI) based on WHO standard in (**a**) deep aquifer and (**b**) shallow aquifer

The spatial distribution of the water quality based on the three indices (EWQI, WQI, and ImpWQI) is shown in Fig. 15.

For the both aquifers, the ImpWQI method shows the best result; it indicates that the Na-Cl water type coincides with the poor, and the extremely poor water quality and the two other indices (WQI and EWQI) indicate good to poor water types. These results reflect the effect of the parameter’s weight in the calculation of the water quality index.

#### Irrigation purposes

The collected samples were assessed for irrigation uses using different indices; the results are illustrated in the Table 7. According the TH (total hardness) values, all samples of the both aquifers present a soft water (TH < 75).

**Table 7:** Irrigation quality indices of Hajeb Layoun-Jelma aquifers

Range	Reference	Classification	Shallow + springs samples		Deep samples	
			Number of samples	% of samples	Number of samples	% of samples
Total hardness (TH)						
< 75	Todd (1980)	Soft	All samples	100%	All samples	100%
75–150		Moderately hard	-	-	-	-
150–300		Hard	-	-	-	-
> 300		Very hard	-	-	-	-
EC (μs/cm)						
< 250	Richard (1954)	Excellent	-	-	-	-
250–750		Good	-	-	2	14%
750–2000		Permissible	3	21%	9	65%
2000–3000		Doubtful	7	50%	1	7%
> 3000		Unsuitable	4	29%	2	14%
Percent sodium (Na%)						
< 20	Wilcox (1955)	Excellent	-	-	-	-
20–40		Good	-	-	3	21%
40–60		Permissible	10	71%	8	58%
60–80		Doubtful	4	29%	3	21%
> 80		Unsafe	-	-	-	-
Alkalinity hazard (SAR)						
< 10	Richard (1954)	Excellent	All samples except PS2	93%	All samples except F13	93%
10–18		Good	1	7%	1	7%
18–26		Doubtful	-	-	-	-
> 26		Unsuitable	-	-	-	-
Magnesium hazard (MH)						
> 50	Raghunath (1987)	Unsuitable	All samples	100%	All samples	100%
< 50		Suitable	-	-	-	-
Permeability index PI						
< 25	Doneen (1964)	Suitable	-	-	-	-
25–75		Moderate				
> 75		Unsuitable	All samples	100%	All samples	100%
Kelley ratio (KR)						
< 1	Kelly (1951)	Suitable	3	21%	3	21%
1–2		Moderate	10	72%	10	72%
> 2		Unsuitable	1	7%	1	7%

The EC values of HJB are ranked into various categories for the both aquifers (shallow and deep aquifer). For the deep aquifer, 79% of samples present good to permissible water quality and 21% of samples indicated a doubtful water class (samples with Na-Cl water type). For the shallow aquifer, 21% of samples are permissible; 79% of samples present doubtful to unsuitable water class (including samples with Na-Cl water type). The %Na indicated that only 71% shallow samples are permissible for irrigation; the %Na of samples with Na-Cl water type varies from 54.3 to 76.71 indicating permissible to doubtful water quality. For the deep samples, three samples (F7, F9, and F10) present a good water class which coincide with the Ca-Cl and Ca-Mg-Cl water type; 58% (Na-Cl water type) indicate permissible water for irrigation, and three samples (Na-Cl water type; F2, F8, and F13) indicate a doubtful water class. The SAR values for HJB samples are ranked into two groups; for the both aquifers, all samples have a low degree of alkalinity hazards (2 < SAR < 10), except three samples with a high alkalinity hazards (10 < SAR < 18). Based only on the SAR values, the samples of HJB are distributed on two water classes (“excellent” to “good”) and its can be utilized for most types of soil. According the calculated values of MH (magnesium hazard) and the PI (permeability index), all samples of the shallow, springs, and deep aquifers are unsuitable for irrigation. The calculated values of Kr show that the groundwater samples of HJB, with Na-Cl water type, are more than 1, indicating moderate to unsuitable water quality for irrigation uses. Based on the seven estimated indices, the most of HJB’s samples are unsuitable for irrigation uses which the shallow samples present an irrigation quality less than the deep samples, and it is due to the shallow aquifer position, the thickness of the vadose zone which has a strong effect on the pollutants infiltration.

#### WILCOX and USSL classification

**Fig. 16:**
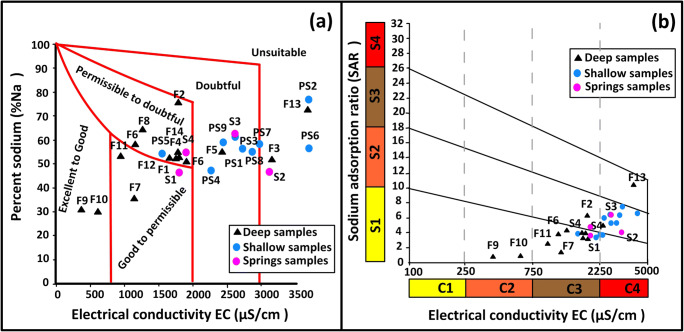
**a** Sodium percentage vs. EC values plot for water quality classification (Wilcox diagram 1955) and **b** USSL classification of HJB samples

The %Na vs. EC values for HJB’s samples were plotted in the Wilcox graphical diagram of irrigation water (Wilcox 1955). The diagram shows that 10 samples present a water quality permissible to doubtful (Na-Cl water type), 3 samples are classed under good to permissible (Ca-Cl and Na-Cl water type), 13 samples are doubtful to unsuitable (Na-Cl water type), and 2 samples are excellent to good (Ca-Cl and Ca-Mg-Cl water type) (Fig. 16a).

The SAR vs. EC values for groundwater samples of HJB were plotted in the USSL diagram of irrigation water (Fig. 16b). Based on USSL diagram (USSL 1954), the water samples show five categories; “C2-S1” (medium salinity with low sodium), “C3-S1” (high salinity with low sodium), “C4-S2” (very high salinity with medium sodium), “C3-S2” (high salinity with medium sodium), and “C4-S3” (very high salinity with high sodium). Based on the combination between EC and SAR, in USSL diagram, HJB have only two deep samples suitable for irrigation (F9 and F10) (medium salinity with low sodium) which coincide with Ca-Cl and Ca-Mg-Cl water type.

## Discussion

The Hajeb Layoun-Jelma basin is the selected site in this research in order to provide its actual water quality situation, with highlights on the water chemistry origins and its suitability (drinking and irrigation). The shallow aquifer shows high salinity in most of the water samples (93% of samples has salinity > 1 g l^−1^ with one sample exceeding 6 g l^−1^) (Fig. 7b). The deep aquifer has moderate salinity: 21% of samples exceeding 1 g l^−1^ and the rest (79%) indicate salinity less than 1 g l^−1^ (Fig. 7a). Groundwater salinity pollution is considered as common Mediterranean problems; it is seen in recent investigations conducted in the shallow aquifers in Northeastern Tunisia (Ghouili et al. 2018) and central-eastern Tunisia (Mnassri et al. 2018). The high level of intake salt in water can cause a serious human health problem (Al Nahian et al. 2018).

In this study, based on Gibbs’s diagram and the inter-parameters correlation, the high salinity levels in the HJB are related to the natural factors (dissolution of carbonates/gypsum and water evaporation). The anthropogenic factors in HJB have also a strong role in the elevation of the salinity concentration such as the increasing number of wells (the number of shallow wells increase from 226 in 1974 to 2328 wells in 2018), the low thickness of the vadose zone (from 3 to 20 m), and the irrigation practices. The huge quantities of fertilizers have an impact on the increasing of rates of Na+ and Cl^−^ (Mnassri et al. 2018). This is showed by the high correlation between Na^+^/Cl^−^ and salinity in this study (Table 3).

The use of modern methods such as EWQI, WQI, and ImpWQI would confer the best understanding of water suitability. Based on the previously mentioned (see the “Water quality indices” section), compared with evaluation results of different weighting methods, it shows that the WQI-based CRITIC weighting method (ImpWQI) is feasible in the HJB’s water quality evaluation. Wang et al. (2018) and Zhang et al. (2020) have applied the improved water quality index method, based on CRITIC weighting, to provide the groundwater’s suitability for drinking purposes. Wang et al. (2018) found that the WQI based on CRITIC weighting (ImpWQI) is the realistic method to assess water quality. As within the HJB, the application of ImpWQI technique shows that for the shallow aquifer, 14 water samples (Table 5 and Fig. 9) range between “poor water” and “extremely poor water,” and for the deep aquifer, the samples range from “excellent water” to “extremely poor water,” for the both aquifer, the “poor” and “extremely poor” water quality coincide with the Na-Cl water type.

The over-abstraction from HJB, the non-treated sewage rejected, and the irrigation practices lead the degradation of HJB’s resources and promote its pollution. To ensure the HJB’s sustainability and avoid the quality problems, it is necessary to improving the irrigation practices by the implementing of a continuously measures to help farmers to adopt the best management practices.

## Conclusion

The HJB has an important economic and social status as a first alternative for sustainable agricultural activities and drinking use for Sidi Bouzid, Kairouan (central Tunisia), and also Sfax (southern coast). The abstraction increases since the mid-1980s and the continuous decline of piezometry make the degradation of the quality and the quantity of this groundwater. To assess the water quality of HJB, 28 water samples were collected in 2017 and analyzed for 11 physico-chemical parameters (temperature, pH, EC, salinity, Na^+^, Ca^2+^, K^+^, Mg^2+^, Cl^−^, HCO_3_^−^, and SO_4_^2−^). For the both aquifers (the MPQ and Beglia aquifers), the order of the abundance of major cations is Na > Mg > Ca > K and anions are Cl > HCO_3_ > SO_4_. The dominant hydrochemical facies, for the shallow aquifer and springs, are Na-Cl and Ca-Mg-Cl; for the deep aquifer, the geochemical facies are Na-Cl, Ca-Mg-Cl, and Ca-Cl. The WQI and the EWQI indicate that most shallow and deep samples present excellent to medium water type and only 7% presents poor water. The ImpWQI present the logic index which indicates 100% and 57% extremely poor water for the shallow and the deep samples, respectively, which coincide with Na-Cl water type. The water quality evaluation for irrigation uses was performed by assembling various geochemistry methods (SAR, TH, % Na, PI, MH, KR, EC). The results indicate that the shallow samples show quality less than the deep one (unsuitability according EC, 79%). The bad irrigation practices, the low thickness, and the high permeability of the vadose zone play a strong role in the infiltration of pollutants and reach to the HJB’s shallow aquifer.
